# Frequency response characteristics and failure model of single-layered thin plate rock mass under dynamic loading

**DOI:** 10.1038/s41598-022-23792-8

**Published:** 2022-11-09

**Authors:** Feng Li, Chenchen Wang, Runchuan Sun, Guangyou Xiang, Baorui Ren, Zhao Zhang

**Affiliations:** 1 School of Emergency Management and Safety Engineering, China University of Mining and Technology (Beijing), Ding No.11, Xueyuan Road, Haidian District, Beijing, 100083 China; 2 State Key Laboratory for Geomechanics and Deep Underground Engineering, China University of Mining and Technology (Beijing), Beijing, 100083 China

**Keywords:** Petrology, Petrology, Natural hazards, Coal

## Abstract

In underground engineering, disturbance of dynamic load can change layered rock mass stress state and induce accidents. Traditional elastic mechanics can’t effectively solve the complex deformation problem. However, Hamiltonian mechanics system can overcome this problem. Dual variables are introduced in symplectic space to solve the deflection equations of single-layered thin plate rock mass. Comparing vibration parameters, it’s found the 1st, 5th and 6th order are effective vibration modes. The resonance characteristics of thin plate are obtained with three dynamic loads. It’s found the thin plate is most likely to resonate and damage due to the smallest resonance frequency interval and the largest vibration amplitude by impact wave and rectangular wave respectively. Then, the vibration mode of multi-layered rock mass is analyzed through Multiple Reference Impact Testing. The failure of fine sandstone is caused by the resonance of effective vibration modes by hammer excitation. Finally, the failure mechanism of thin plate is obtained by the failure theory and LS-DYNA. It’s found the four sides and corners suffer tensile shear failure and shear failure respectively. When tensile failure occurs in central, the main crack and secondary crack propagate along long axis and short axis to form “O-**十**” failure mode.

## Introduction

In the process of geological deposition, sedimentary rocks with bedding structure are formed due to gravity. The sedimentary rocks with obvious bedding structure can also be considered as multi-layered rock mass^1–4^. The layered rock mass can maintain good mechanical properties and strong stability without external disturbance^5,6^, but with the development of underground engineering, the stress state of deep buried layered rock mass will change and cause deformation and failure^7–9^. Therefore, the study on mechanical properties of layered rock mass is urgent. Multi-layered rock mass can be regarded as formed by cementation of single rock mass with various properties. So, the material properties of layered rock mass are heterogeneous, which leads its nonlinear mechanical properties^10,11^. In order to clarify the mechanical properties of layered rock mass, several scholars have carried out a series of mechanical experiments. Zuo prepared coal-rock combination specimens with different lithologies by adhesive tape^12–14^, they established nonlinear theoretical model of coal-rock combination, pre-peak and post-peak stress–strain models under uniaxial compression^15,16^. Zhang discussed relationship between layers and mechanical parameters of multi-layered rock mass by true triaxial compression tests. It was found the peak strength and peak strain increases along with confining pressure increases. The mechanical properties and statistical damage constitutive model under hydraulic-mechanical coupling rock mass was established by comparing the deformation characteristics and failure modes^2^. Wang studied the effect of interlayer thickness and strength on mechanical behavior and failure processes of layered rock mass with holes through uniaxial compression experiments. They found interlayer thickness and strength would lead the change of peak strength and elastic modulus of rock mass^17^. Liu conducted true triaxial compression experiments on two types of foliation orientation according to the large anisotropic deformation caused by the change of original rock stress state. They found the strength and failure modes of layered rocks greatly due to the lateral stress differences (${\sigma }_{2}-{\sigma }_{3}$) has great influence on different foliation directions. Correspondingly, in order to avoid large anisotropic deformation, the angle between the tunnel axis and foliation strike should be as large as possible^18^. Cai considered the influence of intermediate principal stress on rock fracture and strength, the developed path of microcracks and fractures in rock was analyzed by using numerical simulation (FEM/DEM). Stress-induced fracturing and microcracking leading to onion-skin fractures, spalling and slabbing in layered rock mass were revealed^19^. In order to study mechanical properties of composite rock mass, Zienkiewicz proposed multilayered rock mass model which considered viscoplastic strain rate as the sum of each joint group and rock material^20^. Further Wu proposed an anisotropic composite model based on the Drucker-Prager criterion. The model not only can describe anisotropic characteristics of rock strength and deformation, but also can realize nonlinear operation^21^. Several researchers studied rock mechanical properties and failure modes through uniaxial or triaxial compression experiments from macroscopic perspective, which is not comprehensive for considering the mesoscopic evolution of the creep fracture in layered rock mass. Zhao obtained the influence of inclination, thickness, weak layers on creep failure mode through analyzing the initiation, propagation, penetration process of cracks in layered rock mass^22^. Wang studied two curved failure mechanisms for single and multiple rock layers in high-pressure gas storage tunnel based on the upper bound theorem and variational principle. The analytical solutions of critical uplift pressure and failure surface were solved, which can provide theoretical references for tunnels design and construction^23^. By analyzing the stress–strain characteristics and failure characteristics of layered rock mass in these researches, they established the damage constitutive model which can provide strong support for deformation and failure of roadway surrounding rock^4,24,25^. However, the low strain rate loading condition is not suitable for all cases. In roadway excavation and tunnel excavation, the dynamic impact load with high strain rate can be generated when using mechanical tools and blasting excavation^26,27^. The rock failure modes caused by the two loading conditions are quite different, the fundamental reason is the rock failure mechanisms under the two loading conditions are different. In underground engineering, layered rock mass will be damaged by impact load with high strain rate, so it is necessary to study the deformation and failure characteristics of layered rock mass under dynamic impact load.

Because the depletion of coal resources in shallow strata, coal mines enter deep strata mining. With the depth increase, the ground stress and gas stress of coal seam increase. The stress state of layered rock mass became more complicated, dynamic disasters such as gas outburst and rock burst occur more frequently^28–30^. Under strong dynamic disturbances, the damage of multi-layered rock mass in the upper part of the roadway is fatal to the safety production in mines^27,31,32^. Now most failure mode studies of layered rock mass are focusing on the quasi-static loading conditions with low strain rate, while the failure modes under impact load and quasi-static load are quite different^33^. But there are few studies on the layered coal rock mass under impact load, which have great significance for dynamic disaster control in mine. Braunagel studied the effect of rapid stress cycles on dynamic compressive strength using modified split Hopkinson pressure bar (SHPB). They found the failure mode of rock changes from localized failure along discrete fractures to distributed fracturing, the compressive strength of granite decreases twice under cyclic loading^34^. Xie constructed the dynamic mechanical constitutive model of the coal-rock combination specimens by SHPB. They found the strain softening effect were stronger than the hardening effect in coal-rock combination specimens, main damage location was in the coal body^35^. According to the experiment result, Wen studied the dynamic compression characteristics of layered rock mass with significant strength changes. They found the dynamic compressive strength of layered rock mass increases approximately linearly with loading strain rate increased. Recording crack propagation paths by high-speed camera, it was found the bedding plane dip angle control the failure mode for parallel or near-parallel to the dynamic wave trans-mitting direction^36^.Han found the localized slabbing degree of composite rock mass was sensitive to the filled joint thickness, but all specimens ultimately exhibited an axial splitting failure^37^. Furthermore, Han studied the influence of interlayer strength on stress propagation, crack propagation mechanism and failure mode in composite rock-mortar specimens by SHPB and DIC. It was determined that tensile cracks initiate at the rock-mortar interface along the loading direction eventually leading to tensile failure^38^. Qiu studied the influence of interfacial roughness and loading rates effect on crack extension velocity. The results showed that the larger the interfacial roughness, the easier the crack penetrates the composite rock mass, and the average crack propagation speeds increase with loading rate^39,40^. Zheng proposed a numerical 3D mesoscopic approach based on the discrete element method combined with XCT images to characterize the dynamic impact behavior of heterogeneous coal-rock. According to the model, the meso-damage mode and fracture mechanism of heterogeneous coal-rock under different impact modes and impact velocities were studied^41,42^. Since the existing phase field mode can only model the tensile-induced fracture which can’t well reproduce the diversity of dynamic fracture, Duan proposed a new phase field model which involved all the commonly seen dynamic fracture mechanisms to reflect dynamic rock diversity under impact loading. Through this model, crack initiation and propagation can be automatically characterized by phase field evolution equation, and different fracture modes of layered rock can be predicted^43^. In addition, some scholars used thin plate theory to study the breaking form and caving law of the gob roof. Zuo simulated the roof fracture experiment of goaf by thin plates, which explained the O–X failure mode of thin plate was initiated by tensile-shear stress^44^. Because of transverse shear stress strengthened with plate thickness increase, the failure mode of “O − *” appear. These studies provide good foundation for exploring the dynamic mechanical properties of layered rock mass, but now the complex deformation characteristics and failure mode of layered rock mass are still unclear using traditional elastic mechanics. However, the failure characteristics of layered rock mass are very important for underground engineering. So, further research on this aspect is required.

Now, the fracture research on thin plates is based on the traditional elastic mechanics, it’s difficult to find all solutions which strictly meet the boundary conditions in the solution process. So, the boundary conditions are often given by saint venant principle^45–47^. However, the substitution method will lead large errors in the boundary calculation results, which is difficult to reflect the real situation^48,49^. Hamilton mechanical system can overcome the shortcomings of some boundary conditions in traditional elastic mechanics^50–52^. During solving process, this method can reduce the order of high-order differential equations to decrease the difficulty of solving by introduce the dual variables in symplectic space^53,54^.So the Hamiltonian mechanics system can solve the end, local complex deformation problems which are difficult to deal by traditional elastic mechanics^55^. In order to clarify the failure characteristics of multi-layered rock mass, it’s necessary to clarify the failure characteristics of single-layered thin plate rock mass. In this paper, the mechanical problem of single-layered thin plate rock mass is transformed into solving the eigenvalue and eigenvector of Hamiltonian. Based on the separation variable method, the deflection vibration mode function of single-layered thin plate rock mass is derived, the controlling equations of thin plate are solved by the Duhame integral. Subsequently, the resonance characteristics of single-layered thin plate rock mass under different dynamic loads are discussed, which the correctness of the theoretical derivation is verified by analyzing the effective vibration mode parameters in MRIT. Finally, the failure characteristics of single-layered thin plate rock mass under impact load are obtained through failure theory and numerical simulation.

## Deflection equation of single-layered thin plate rock mass under forced vibration

### Control equation of single-layered thin plate rock mass

**Figure 1:**
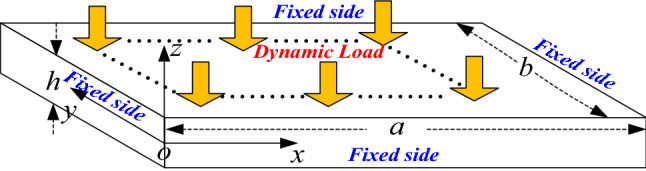
Mechanical calculation model of single layered thin plate rock mass.

As shown in Fig. 1, in this paper the single-layered thin plate rock mass is transversely isotropic in mechanical properties. The density of the rock mass is *ρ*, the thickness is *h*, the size is *a* × *b*, the elastic modulus is *E*, the Poisson's ratio is υ, the x–y plane is neutral plane.

Based on the transient equilibrium conditions of the internal mechanics of thin plate rock mass, the differential equation of single-layered thin plate rock mass can be derived under forced vibration^56^:

1 $$ D\nabla^{4} w(x,y,t) + \rho h\frac{{\partial^{2} w(x,y,t)}}{{\partial t^{2} }} = q(t) $$

where *w* (*x*, *y*, *t*) is the deflection; *D* is the bending stiffness of single-layered thin plate rock mass; *ρ* is the density of single-layered thin plate rock mass; *q*(*t*) is external dynamic load.

To solve the homogeneous Eq. (1), set *q*(*t*) = 0, the free vibration differential equation of single-layered thin plate rock mass as follows^50^:

2 $$ D\nabla^{4} w(x,y,t) + \rho h\frac{{\partial^{2} w(x,y,t)}}{{\partial t^{2} }} = 0 $$

In this paper, we assume that the vibration of single-layered thin plate rock mass has the following harmonic oscillator with time as:

3 $$ w(x,y,t) = W(x,y)e^{iwt} $$

where ω is the natural frequency; *W*(*x*,*y*) is the deflection mode function.

Bring Formula (3) into Formula (2), we can get:

4 $$ \frac{{\partial^{4} W}}{{\partial^{4} x}} + \frac{{\partial^{4} W}}{{\partial^{2} x\partial^{2} y}} + \frac{{\partial^{4} W}}{{\partial^{4} y}} = k^{4} W $$

where *k*^4^ = *ρh*ω^2^/D.

### Hamilton dual vibration equation of single-layered thin plate rock mass

It is necessary to decouple the physical parameters in (4) using the Hamilton dual equations. If set θ = ∂*W*/∂*x*, the relationship between the physical parameters as follows^57^:

5 $$ \frac{\partial \theta }{{\partial x}} = - \frac{{M_{x} }}{D} - \upsilon \frac{{\partial^{2} W}}{{\partial y^{2} }} $$

6 $$ \frac{{\partial V_{x} }}{\partial x} = D(1 - \upsilon^{2} )\frac{{\partial^{4} W}}{{\partial y^{4} }} - \upsilon \frac{{\partial^{2} M_{x} }}{{\partial y^{2} }} - \rho \omega^{2} W $$

7 $$ \frac{{\partial M_{x} }}{\partial x} = V_{x} + 2D(1 - \upsilon )\frac{{\partial^{2} \theta }}{{\partial y^{2} }} $$

If vector *v* = [*W*, *θ*, − *V*_*x*_, *M*_*x*_], the dual equation in Hamilton system as follows^58^:

8 $$ v^{\prime} = Hv $$

The Eq. (8) can also be expressed as:

9 $$ \left[ {\begin{array}{*{20}c} {\frac{\partial W}{{\partial x}}} \\ {\frac{\partial \theta }{{\partial x}}} \\ {\frac{{\partial ( - V_{x} )}}{\partial x}} \\ {\frac{{\partial M_{x} }}{\partial x}} \\ \end{array} } \right] = \left[ {\begin{array}{*{20}c} 0 & 1 & 0 & 0 \\ { - \upsilon \frac{{\partial^{2} }}{{\partial y^{2} }}} & 0 & 0 & { - \frac{1}{D}} \\ { - D(1 - \upsilon^{2} )\frac{\partial 4}{{\partial y4}} + \rho \omega^{2} h} & 0 & 0 & {\upsilon \frac{{\partial^{2} }}{{\partial y^{2} }}} \\ 0 & {2D(1 - \upsilon )\frac{{\partial^{2} }}{{\partial y^{2} }}} & {{ - }1} & 0 \\ \end{array} } \right] * \left[ {\begin{array}{*{20}c} W \\ \theta \\ { - V_{x} } \\ {M_{x} } \\ \end{array} } \right] $$

### Solution of the Hamilton dual equations

Based on the symplectic geometry method and separation of variables, the solutions of (9) can be obtained. *W*(*x*) and *W*(*y*) are the deflection modes along the *x* and *y* directions respectively, the specific forms as follows^58–60^:

10 $$ W(x) = a_{1} e^{{i\beta_{1} x}} + b_{1} e^{{ - i\beta_{1} x}} + c_{1} e^{{\beta_{2} x}} + d_{1} e^{{ - \beta_{2} x}} = A_{1} \cos \beta_{1} x + B_{1} \sin \beta_{1} x + C_{1} \cosh \beta_{2} x + D_{1} \sinh \beta_{2} x $$

11 $$ W(y) = a_{2} e^{{i\alpha_{1} y}} + b_{2} e^{{ - i\alpha_{1} y}} + c_{2} e^{{\alpha_{2} y}} + d_{2} e^{{ - \alpha_{2} y}} = A_{2} \cos \alpha_{1} y + B_{2} \sin \alpha_{1} y + C_{2} \cosh \alpha_{2} y + D_{2} \sinh \alpha_{2} y $$

where *α*_1_ and *α*_2_ are the eigenvalues in the *x* direction, *β*_1_ and *β*_2_ are eigenvalues in the *y* direction, *α*_1_, *α*_2_, *β*_1_, *β*_2_ satisfy the following rules:

$$\alpha_{1}^{2} + \alpha_{2}^{2} = \beta_{1}^{2} + \beta_{2}^{2} = 2k^{2}$$, $$\alpha_{1}^{2} + \beta_{1}^{2} = k^{2}$$, $$\alpha_{2}^{2} + \beta_{2}^{2} = 3k^{2}$$, $$\alpha_{2}^{2} - \beta_{1}^{2} = k^{2}$$.

### Deflection equation of single-layered thin plate rock mass under free vibration

In order to obtain the deflection equation of single-layered thin plate rock mass under free vibration, the boundary conditions should be determined. In this section, single-layered thin plate rock mass in a state of four edges fixed and isn’t disturbed by external load.

In the y direction of thin plate rock mass, there are the following relations:

*W*(*x*,0) = 0, ∂*W*(*x*,0)/∂y = 0; *W*(*x*,b) = 0, ∂*W*(*x*,b)/∂y = 0.

Combined with (9), we can get:

12 $$ \left[ {\begin{array}{*{20}c} 1 & 0 & 1 & 0 \\ 0 & {\alpha_{1} } & 0 & {\alpha_{2} } \\ {\cos \alpha_{1} b} & {\sin \alpha_{1} b} & {\cosh \alpha_{2} b} & {\sinh \alpha_{2} b} \\ { - \alpha_{1} \sin \alpha_{1} b} & {\alpha_{1} \cos \alpha_{1} b} & {\alpha_{2} \sinh \alpha_{2} b} & {\alpha_{2} \cosh \alpha_{2} b} \\ \end{array} } \right] * \left[ {\begin{array}{*{20}c} {A_{2} } \\ {B_{2} } \\ {C_{2} } \\ {D_{2} } \\ \end{array} } \right] = \left[ {\begin{array}{*{20}c} 0 \\ 0 \\ 0 \\ 0 \\ \end{array} } \right] $$

With (11), the frequency equation along the *y* direction can be obtained as:

13 $$ \frac{{1{ - }\cos \alpha_{1} b\cosh \alpha_{2} b}}{{\sin \alpha_{1} b\sinh \alpha_{2} b}} = \frac{{\alpha_{1}^{2} - \alpha_{2}^{2} }}{{2\alpha_{1} \alpha_{2} }} $$

The deflection equation as follows:

14 $$ \begin{gathered} W(x) = - \cos \beta_{1} x + \frac{{\beta_{2} }}{{\beta_{1} }}k_{2} \sin \beta_{1} x + \cosh \beta_{2} x - k_{2} \sinh \beta_{2} x, \hfill \\ W(y) = - \cos \alpha_{1} y + \frac{{\alpha_{2} }}{{\alpha_{1} }}k_{1} \sin \alpha_{1} y + \cosh \alpha_{2} y - k_{1} \sinh \alpha_{2} y \hfill \\ \end{gathered} $$

where

$$k_{1} = \frac{{\cos \alpha_{1} b - \cosh \alpha_{2} b}}{{\frac{{\alpha_{2} }}{{\alpha_{1} }}\sin \alpha_{1} b - \sinh \alpha_{2} b}}$$, $$k_{2} = \frac{{\cos \beta_{1} a - \cosh \beta_{2} a}}{{\frac{{\beta_{2} }}{{\beta_{1} }}\sin \beta_{1} a - \sinh \beta_{2} a}}$$.

### Deflection equation of single-layered thin plate rock mass under forced vibration

#### Solution of the control equation

The solutions’ form of the non-homogeneous control Eq. (1) can be expressed as follows:

15 $$ w(x,y,t) = \sum\limits_{n = 1}^{\infty } {W_{n} (x,y)\varphi_{m} (t)} $$

Insert (15) into (1):

16 $$ \sum\limits_{m = 1}^{\infty } {[D\nabla^{4} W_{m} (x,y)} \varphi_{m} (t) + \rho hW_{m} (x,y)\varphi_{m} ^{\prime\prime}(t)] = q(t) $$

With (4),

17 $$ D\nabla^{4} W_{m} (x,y) = \rho h\omega_{m}^{2} W_{m} (x,y) $$

Then,

18 $$ \sum\limits_{m = 1}^{\infty } {\rho hW_{m} (x,y)} [\omega_{m}^{2} \varphi_{m} (t) + \varphi_{m} ^{\prime\prime}(t)] = q(t) $$

The orthogonality of the deflection equations as follows^61^:

19 $$ \iint\limits_{\Omega } {\rho hW_{m} (x,y)W_{n} (x,y)}dxdy = 0,(m \ne n) $$

We multiply both sides of (18) by *W*_*m*_(*x*,*y*) and do integral over the thin plate:

20 $$ \iint\limits_{\Omega } {\rho hW^{2}_{m} (x,y)}[\omega_{m}^{2} \varphi_{m} (t) + \varphi_{m} ^{\prime\prime}(t)]dxdy = \iint\limits_{\Omega } {q(t)W_{m} (x,y)}dxdy $$

Set

21 $$ M_{m} = \iint\limits_{\Omega } {\rho hW_{m}^{2} (x,y)}dxdy,P_{m} (t) = \iint\limits_{\Omega } {q(t)W_{m} (x,y)}dxdy $$

Then,

22 $$ \varphi_{m} ^{\prime\prime}(t) + \omega_{m}^{2} \varphi_{m} (t) = \frac{1}{{M_{m} }}P_{m} (t) $$

Based on Duhamel’ Integral, the solutions can be expressed as follows:

23 $$ \varphi_{m} (t) = \frac{1}{{M_{m} \omega_{m} }}\int\limits_{0}^{t} {P_{m} (\tau )} \sin \omega_{m} (t - \tau )d\tau $$

And then,

24 $$ \varphi_{m} (t) = \frac{{\iint\limits_{\Omega } {W_{m} (x,y)}dxdy}}{{M_{m} \omega_{m} }}\int\limits_{0}^{t} {q(\tau )} \sin \omega_{m} (t - \tau )d\tau $$

#### The main vibration mode *W*_*m*_*(x,y)* of single-layered thin plate rock mass

Combining the frequency equation of single-layered thin plate rock mass, the main vibration mode is solved by Newton's iterative method and the calculated values of the first 10 order vibration modal parameters are shown in Table 1. It’s found that the 1st order,5th order and 6th order vibration functions are the main vibration modes by comparing the first 10 order vibration modal parameters (Table 2).

**Table 1:** The first 10 vibration modal parameters of single-layered thin plate rock mass.

Parameter	1st order	2nd order	3rd order	4th order	5th order	6th order	7th order	8th order	9th order	10th order
*β*_1_	1.443	1.301	2.573	2.483	1.227	3.643	2.404	3.589	2.346	2.346
*β*_2_	2.145	3.247	2.949	3.765	4.437	3.895	4.802	4.514	5.930	5.930
*α*_1_	1.122	2.104	1.019	2.001	3.015	0.975	2.940	1.935	3.904	3.851
*α*_2_	2.329	2.795	3.778	4.042	3.479	5.243	4.494	5.433	4.248	5.083
*k*	1.828	2.474	2.767	3.189	3.255	3.771	3.797	4.078	4.079	4.509
$\iint\limits_{\Omega } {W_{m} (x,y)}dxdy$	13.589	8.1 × 10^–12^	7.0 × 10^–12^	0	8.503	12.565	− 4.3 × 10^–11^	3.1 × 10^–9^	4.9 × 10^–10^	0

**Table 2:** Main mode frequency of single-layered thin plate rock mass with different lithology (*ω*_m_).

Frequency Lithology	1st order (rad·s^−1^/Hz)	5st order (rad·s^−1^/Hz)	6st order (rad·s^−1^/Hz)	Elastic modulus E(GPa)	Poisson ratio	Density (Kg/m^3^)	Size
Fine sandstone	189.4/30.1	600.6/95.6	806.3/128.3	28.8	0.2	2800	*a*:3.6; *b*:3.0;
Sandstone	137.5/21.9	436.1/69.4	585.3/93.1	13.5	0.25	2550	
Coarse sandstone	135.6/21.6	429.9/68.4	577.0/91.8	14.1	0.22	2700	
Limestone	115.0/18.3	364.7/58.0	489.5/77.9	10.7	0.18	2800	
Siltstone	114.7/18.2	363.6/57.9	488.0/77.7	10.1	0.2	2680	
Sandy mudstone	105.5/16.8	334.6/53.3	449.2/71.5	7.8	0.27	2530	*h*:0.06
Mudstone	91.8/14.6	291.1/46.3	390.8/62.2	5.8	0.28	2500	
Coal	63.2/10.1	200.5/31.9	269.1/42.8	1.5	0.32	1400	
Soft Coal Seams	34.9/5.6	110.5/17.6	148.4/23.6	0.4	0.39	1300	

## Vibration laws of single-layered thin plate rock mass under dynamic loading

### The Fourier series expressions under dynamic loading

Fourier transform can be used to obtain the Fourier series expressions and waveforms of rectangular waves, triangular wave and impact wave. The Fourier series expressions and waveforms of dynamic loading with amplitude A = 1, period T = 2 s as shown in Table 3. When the Fourier series expressions are inserted in Eq. (4), the time harmonic vibration term *φ*_m_(t) of thin plate rock mass as shown in Table 4. Then, the vibration equation of thin plate rock mass can be obtained with Eq. (3). When the vibration frequency of dynamic loading and the main vibration mode (*ω*_m_) of thin plate rock mass satisfy Eq. (25), resonance phenomenon occurs.

25 $$ n^{{2}} \omega^{{2}} = \omega_{{\text{m}}}^{{2}} ,{\text{ while:}}\;n\omega = \omega_{{\text{m}}} \left( {n = {1},{ 2},{ 3},{ 4} \ldots } \right) $$

**Table 3:** The Fourier series expressions and waveforms of dynamic loading *q*(*t*) (A = 1).

Dynamic loading	FFT series expression	waveforms of dynamic loading
Rectangular wave	$q(t) = \frac{4A}{\pi }\sum\limits_{n = 1}^{\infty } \frac{1}{n} \sin^{2} \left( {\frac{n\pi }{2}} \right)\sin (n\omega t)$	
Triangular wave	$q(t) = \frac{A}{2} + \frac{4A}{{\pi^{2} }}\sum\limits_{n = 1}^{\infty } {\frac{1}{{n^{2} }}} \sin^{2} \left( {\frac{n\pi }{2}} \right)\cos (n\omega t)$	
Impact wave	$q(t) = \frac{A}{n}\left( {\frac{1}{T} + \frac{2}{T}\sum\limits_{n = 1}^{\infty } {\cos (n\omega t)} } \right)$

**Table 4:** The time harmonic vibration term *φ*_m_(t) of single-layered thin plate rock mass under dynamic loading.

Dynamic loading	The time harmonic vibration term *φ*_m_(t)	
Rectangular wave	$\varphi_{m} (t) = \frac{{4AA_{m} }}{\pi }\sum\limits_{n = 1}^{\infty } {\frac{1}{n}\sin^{2} \left( {\frac{n\pi }{2}} \right)\frac{{n\omega \sin (\omega_{m} t) - \omega_{m} \sin (n\omega t)}}{{n^{2} \omega^{2} - \omega_{m}^{2} }}}$	$A_{m} = \frac{{\iint\limits_{\Omega } {W_{n} (x,y)}dxdy}}{{M_{n} \omega_{m} }}$
Triangular wave	$\varphi_{m} (t) = AA_{m} \left[ {\frac{{1 - \cos (\omega_{m} t)}}{{2\omega_{m} }} - \frac{{4\omega_{m} }}{{\pi^{2} }}\sum\limits_{n = 1}^{\infty } {\frac{1}{{n^{2} }}\sin^{2} \left( {\frac{n\pi }{2}} \right)\frac{{\cos (n\omega t) - \cos (\omega_{m} t)}}{{n^{2} \omega^{2} - \omega_{m}^{2} }}} } \right]$	
Impact wave	$\varphi_{m} (t) = \frac{{AA_{m} }}{\pi }\left[ {\frac{{1 - \cos (\omega_{m} t)}}{{{{2n\omega_{m} } \mathord{\left/ {\vphantom {{2n\omega_{m} } \omega }} \right. \kern-\nulldelimiterspace} \omega }}} - \frac{{\omega \omega_{m} }}{n}\sum\limits_{n = 1}^{\infty } {\frac{{\cos (n\omega t) - \cos (\omega_{m} t)}}{{n^{2} \omega^{2} - \omega_{m}^{2} }}} } \right]$	

### Resonance frequency distribution laws of dynamic loading

The vibration frequency and period of the dynamic load is *ω* = π rad/s (0.5 Hz), T = 2 s. By plotting *φ*_m_(t) versus *ω*_m_ under three different dynamic loads, the resonant frequency characteristics of thin plate rock mass are revealed at t = 0–20 s, 0.01 s interval, algebraic and term n = 150.

#### Resonance frequency distribution laws of rectangular wave

**Figure 2:**
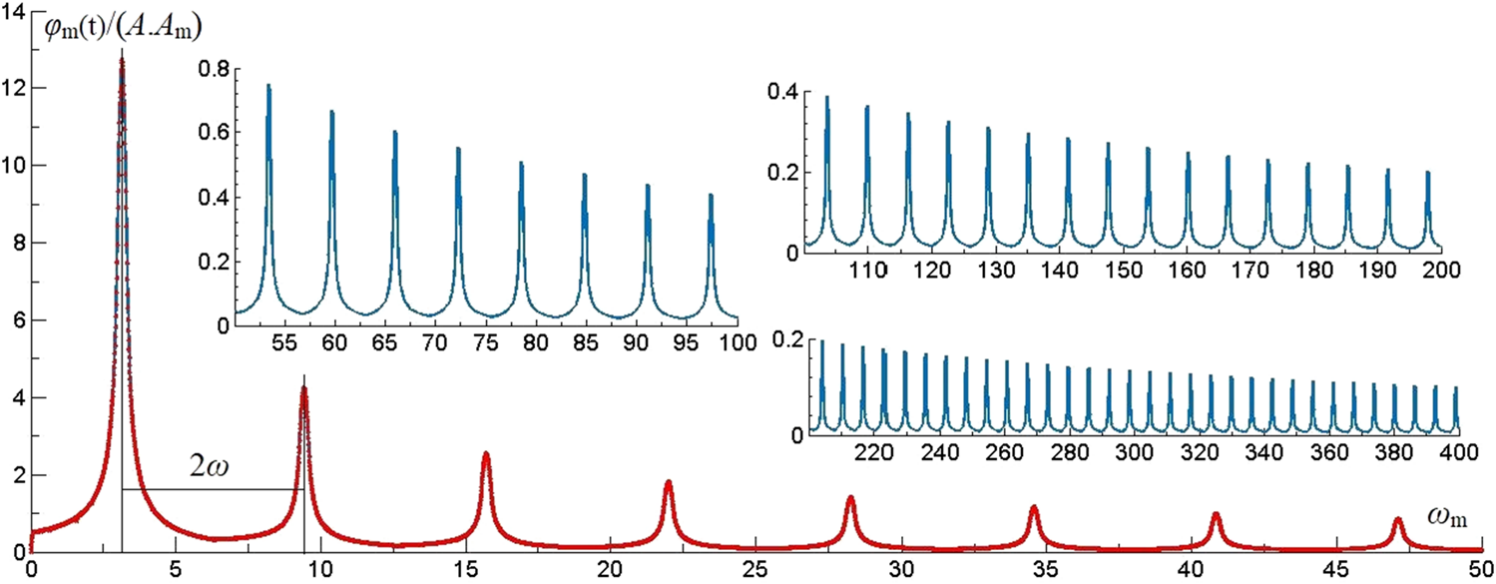
The vibration of *φ*_m_(t) of *ω*_m_ under rectangular wave.

Under the rectangular wave, the time harmonic vibration term *φ*_m_(t) of effective modes which the 1st, 5th and 6th orders are analyzed and the vibration patterns as shown in Fig. 2. It’s found that the resonance frequency interval caused by rectangular wave is 2π (2*ω*), the resonant amplitude *φ*_m_(t) distributed in *ω*_m_ = π–15π rad/s (*ω–*15*ω*). According to (25), the thin plate rock mass will resonate when *ω*_m_ = *nω*. With rectangular wave, when n is odd number, the resonance phenomenon is obvious. When n is even number, the resonance phenomenon is not obvious. The amplitude of resonance decreases exponentially with the resonance frequency increases.

#### Resonance frequency distribution laws of triangular wave

**Figure 3:**
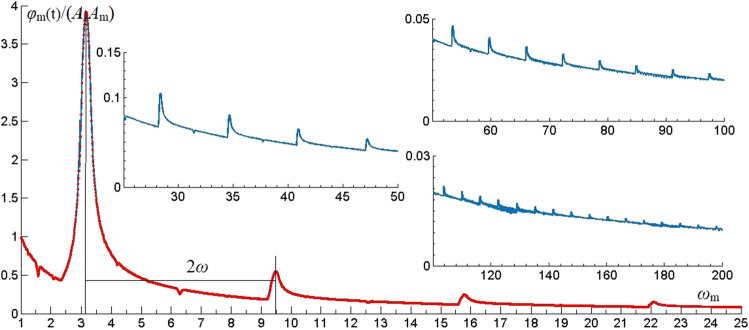
The vibration of *φ*_m_(t) of *ω*_m_ under triangular wave.

Under the triangular wave, the time harmonic vibration term *φ*_m_(t) of effective modes which the 1st, 5th and 6th orders are analyzed and the vibration patterns as shown in Fig. 3. Like the effect of rectangular wave, the resonance frequency interval caused by triangular wave is 2π (2*ω*), the resonant amplitude *φ*_m_(t) distributed in *ω*_m_ = π–15π rad/s (*ω–*15*ω*). With triangular wave, when n is odd number, the resonance phenomenon is obvious. When n is even number, the resonance phenomenon is not obvious. The amplitude of resonance decreases exponentially with the resonance frequency increases.

#### Resonance frequency distribution laws of impact wave

**Figure 4:**
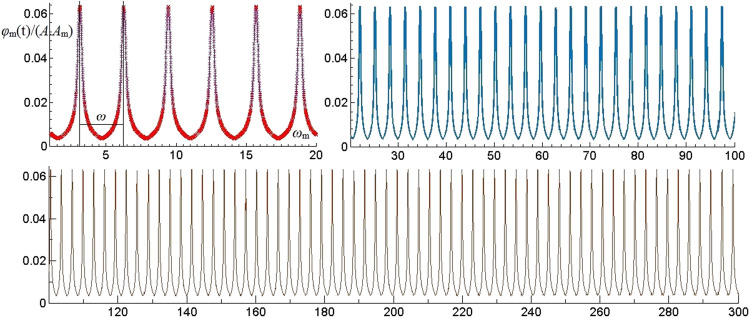
The vibration of *φ*_m_(t) of *ω*_m_ under impact wave.

Under the impact wave, the time harmonic vibration term *φ*_m_(t) of effective modes which the 1st, 5th and 6th orders are analyzed and the vibration patterns as shown in Fig. 4. Different from the rectangular wave and triangular wave, the resonance frequency interval caused by impact wave is π (ω), the resonant amplitude *φ*_m_ (t) relatively small. With impact wave, single-layered thin plate rock mass will resonate when n is positive integer. The amplitude of resonance keeps constant with the resonance frequency increases.

By analyzing the resonance parameters generated from different dynamic loads. On the one hand, the resonance frequency interval caused by impact wave is the smallest in dynamic loads, so that single-layered thin plate rock mass will resonate easily by impact wave. On the other hand, the resonant amplitude of rectangular wave is the biggest in dynamic loads, so that the resonance effect caused by rectangular wave is the most obvious. Correspondingly, the damage effect by rectangular wave acting on thin plate rock mass is the most significant.

### Spectrum structure analysis experiment of dynamic loading

#### Experimental system and sample preparations

The similarity simulation experiment is widely used to simulate the deformation and failure laws of overlying strata in mining. In this paper, the similarity simulation experiment platform is 1800 mm × 160 mm × 1100 mm. We use five types rock mass to simulate multi-layer coal and rock mass, the rock mass from the top to the bottom of the model are fine sandstone, medium sandstone, coal, coarse sandstone, mudstone respectively. The thickness of each rock mass is 200 mm and the bulk density similarity ratio is 1:1.6. The composition and ratio of similar materials in each layer rock mass as shown in Table 5.

**Table 5:** The composition and ratio of similar materials of each layer.

Serial number	Layers of coal and rock mass	Compressive strength/MPa		Fine sand /Kg	Cement/Kg	Gypsum/Kg	Water /Kg
		Raw material	Similar material				
1	Fine sandstone	120	0.75	72.98	7.30	17.03	9.73
2	Medium sandstone	80.1	0.50	81.09	8.11	8.11	9.73
3	Coal	10	0.063	86.49	7.57	3.24	9.73
4	Coarse sandstone	61.5	0.384	83.40	6.95	6.95	9.73
5	Mudstone	27.4	0.171	88.46	6.32	6.32	10.11

#### Action points and sensors distribution

**Figure 5:**
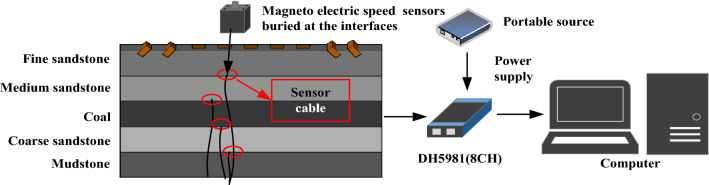
Experimental model and data acquisition system.

In the traditional experimental modal analysis, the force hammer is used as the excitation device. Hammering method is the most widely used modal test method due to its convenient installation, strong mobility and less channel requirements. According to the number of data acquisition equipment channels, hammering method can be divided into Single Reference Impact Testing (SRIT) and Multiple Reference Impact Testing (MRIT)^62,63^. MRIT can obtain more row or column matrix parameters for spectral analysis, MRIT is more convenient than SRIT^64,65^. So MRIT is used in this paper, 9 hammer action points are arranged in turn on the top surface of fine sandstone, 100 mm apart from two adjacent action points. In order to reduce the damage of fine sandstone structure caused by the force hammer, iron blocks with size of 80 mm × 80 mm are placed at each action point as shown in Fig. 5. The 1#–5# action point is arranged vertically at the top plane center of fine sandstone, the action points 6#, 8# and 7#, 9# are located on the left and right sides of the top of fine sandstone, which arranged at 45° and 135° in the horizontal direction. The vibration amplitude which caused by force hammer is measured by magnetoelectric speed sensors (2D001), the No.1-No.4 sensors are distributed respectively at the interfaces of each layer rock mass, the surface of the sensor (signal receiving surface) attached the interface of each rock mass. The magnetoelectric signal is transmitted to the distributed network dynamic signal test system (DH5981), which is used for data acquisition and analysis.

#### Variations amplitude and amplitude-frequency distribution

**Figure 6:**
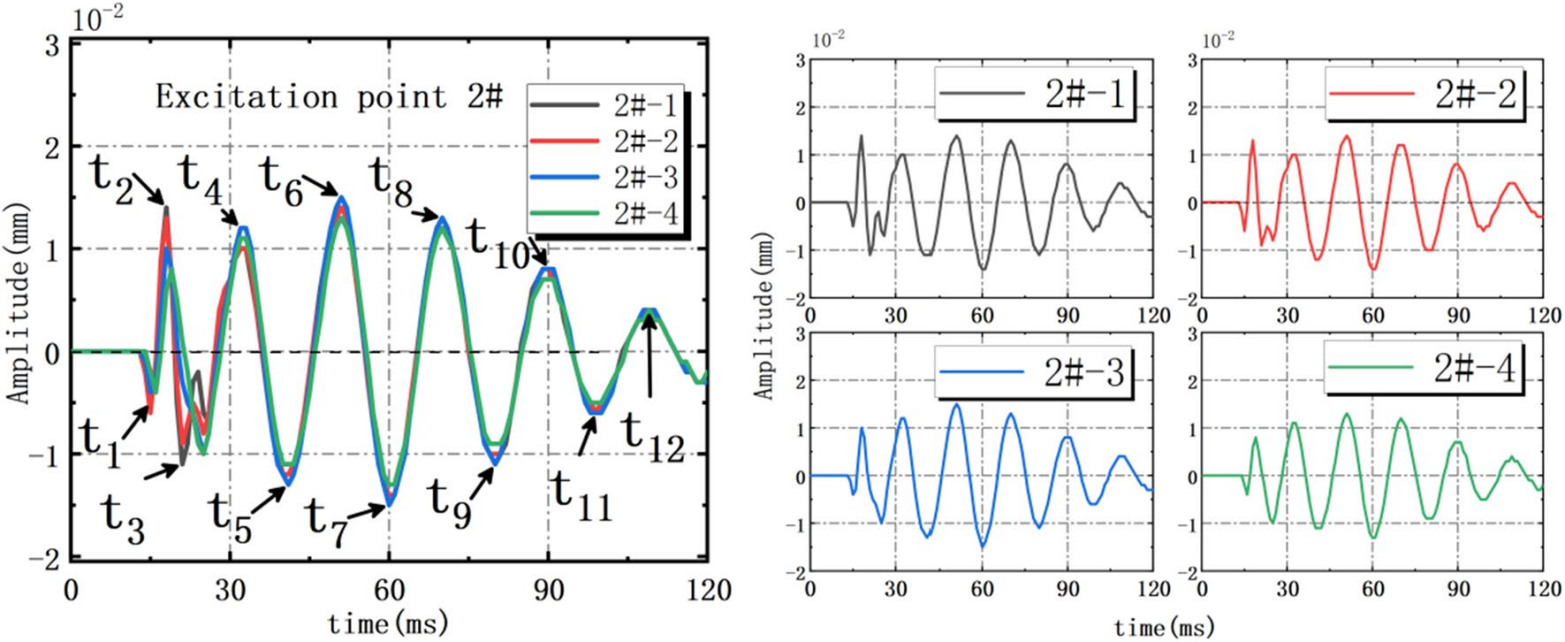
Amplitude time-history curves under 2# action point excited alone.

When 2# action point is acted by the force hammer, the amplitude curve of each layer rock mass obtained by No.1–No.4 sensors as shown Fig. 6. It’s found that, the vibration trends measured by four sensors are similar. When *t*_1_ = 15 ms, the No.1 sensor reaches the first extreme value s_1_ = − 0.005 mm; When *t*_6_ = 51 ms, the No.1 sensor reaches the peak s_6_ = 0.014 mm. When the 2#, 4# and 6# action points are respectively acted by the force hammer, the extreme points and corresponding time points of amplitude curve as shown in Table 6. Under the disturbance of impact load, the vibration period of each layer rock mass is T_1_ = 18–21 ms, the vibration frequency is 45–52.5 Hz.

**Table 6:** The extreme points of amplitude curves of 2#, 4# and 6# action points.

Extreme value (mm) /Time (ms)	*s*_1_*/t*_1_	*s*_2_*/t*_2_	*s*_3_*/t*_3_	*s*_4_*/t*_4_	*s*_5_*/t*_5_	*s*_6_*/t*_6_	*s*_7_*/t*_7_	*s*_8_*/t*_8_	*s*_9_*/t*_9_	*s*_10_*/t*_10_	*s*_11_*/t*_11_	*s*_12_*/t*_12_
2# action point	0.005/15	0.014/18	0.011/21	0.010/33	0.011/42	0.014/51	0.014/61	0.013/70	0.011/80	0.006/99	0.006/99	0.004/109
4# action point	0.004/12	0.009/15	0.007/22	0.012/29	0.013/38	0.015/49	0.015/58	0.014/67	0.011/78	0.008/86	0.007/97	0.004/117
6# action point	0.001/7	0.002/13	0.005/18	0.005/25	0.005/33	0.005/43	0.005/52	0.004/62	0.003/72	0.003/82	0.002/92	0.002/101

**Figure 7:**
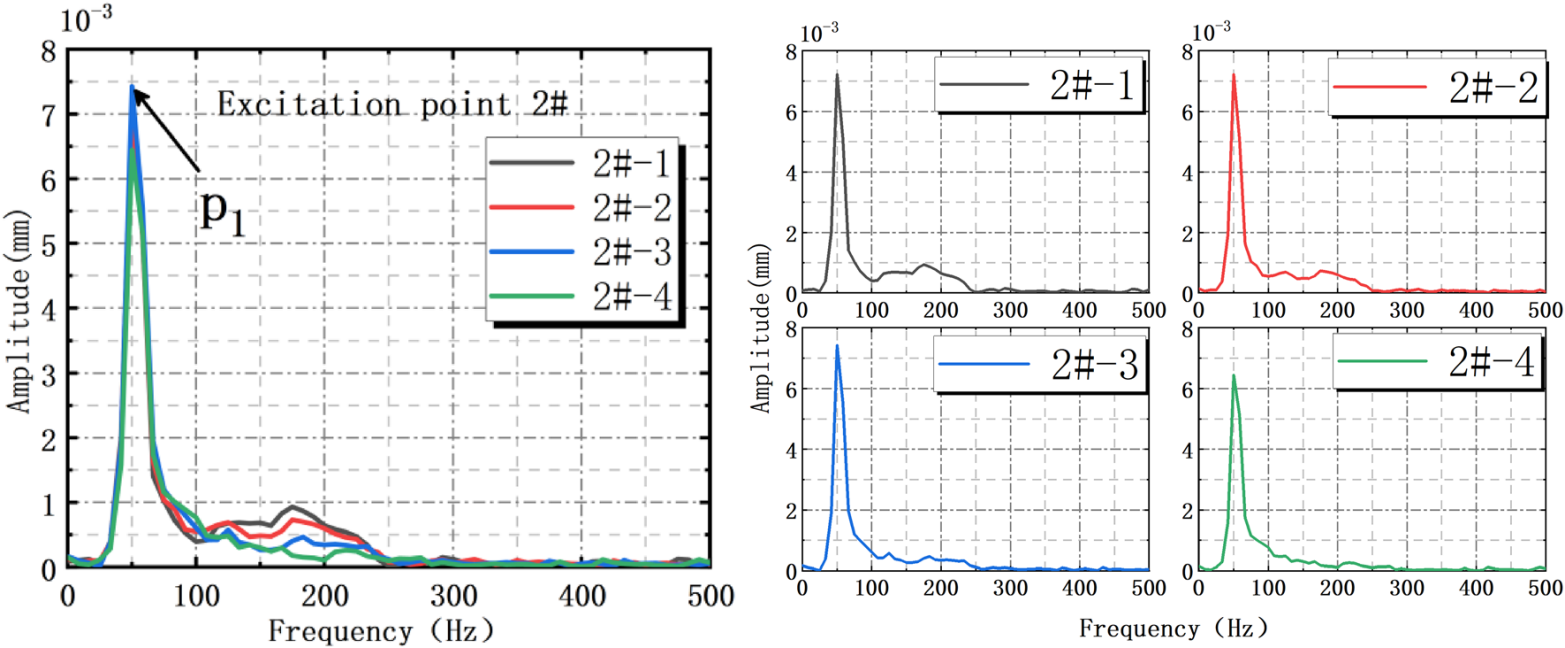
Amplitude-frequency distribution of interface vibration under 2# action point excitation.

When 2# action point is acted by the force hammer, the amplitude-frequency distributions of each sensor as shown Fig. 7. It’s found that the amplitude vibration trends measured by four sensors are similar. When *p*_1_ = 50.5 Hz, the No.1 sensor reaches peak 7.2 × 10^−3^ mm, other sensors reach peak at this nearby frequency. Based on Hilbert Huang transform (HHT), the interface vibration waveforms are decomposed by EEMD^27,29,32^. Combined with energy formula: ${\int }_{-\infty }^{\infty }{x}^{2}(t)dt$, the energy distributions and marginal spectrum of the decomposed waveforms are obtained^30,56^.

**Figure 8:**
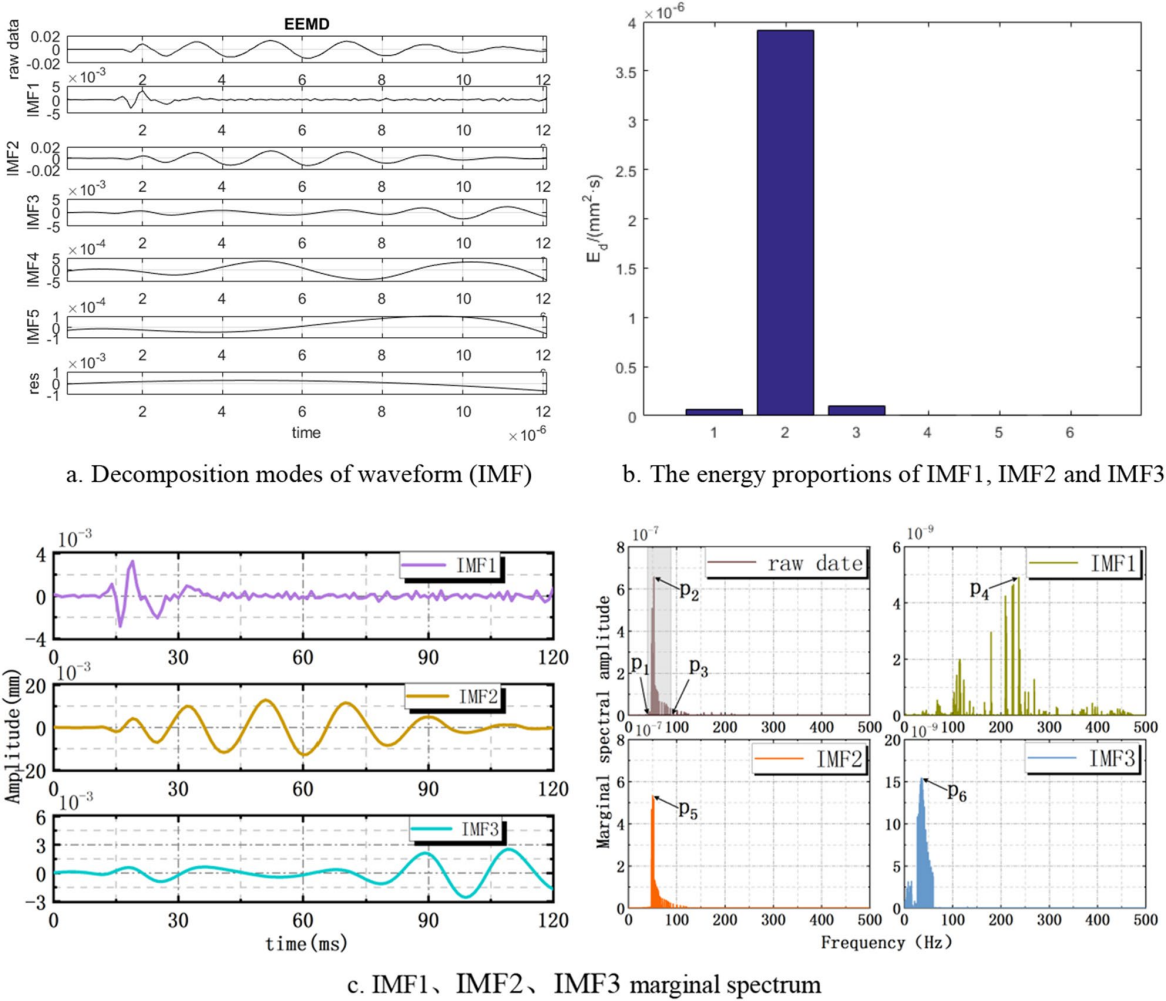
The waveform decomposition under 2# action point excitation.

With 2# action point is excited, the vibration waveform is decomposed into five vibration modes (IMF1–IMF5) as shown in Fig. 8a. IMF1, IMF2 and IMF3 occupy most energy of the vibration waveform in Fig. 8b, so they are called effective vibration modes. IMF2 is called the main vibration mode due to it takes up 96% of the total energy. It’s found that the vibration frequency of the original waveform in 40.0–90.0 Hz (P_1_–P_3_) by analyzing the marginal spectrum in Fig. 8c. When the vibration frequency *P*_2_ = 50.5 Hz, the marginal spectral amplitude reaches the maximum value. By analyzing the marginal spectrum of effective vibration modes (IMF1, IMF2 and IMF3), the predominant frequencies of IMF1、IMF2 and IMF3 are *P*_4_ = 236.8 Hz, *P*_5_ = 50.2 Hz and *P*_6_ = 45.9 Hz respectively. The resonance frequency range caused by impact load is 225–262.5 Hz and 45–52.5 Hz. According to Table 2, it’s found that the main vibration modes frequency of fine sandstone is 30.1–128.3 Hz which is similar to the disturbance frequency of impact load. This cause resonance and enhance the failure effect of fine sandstone under impact load.

#### The failure mode of single-layered thin plate rock mass under dynamic loading

**Figure 9:**
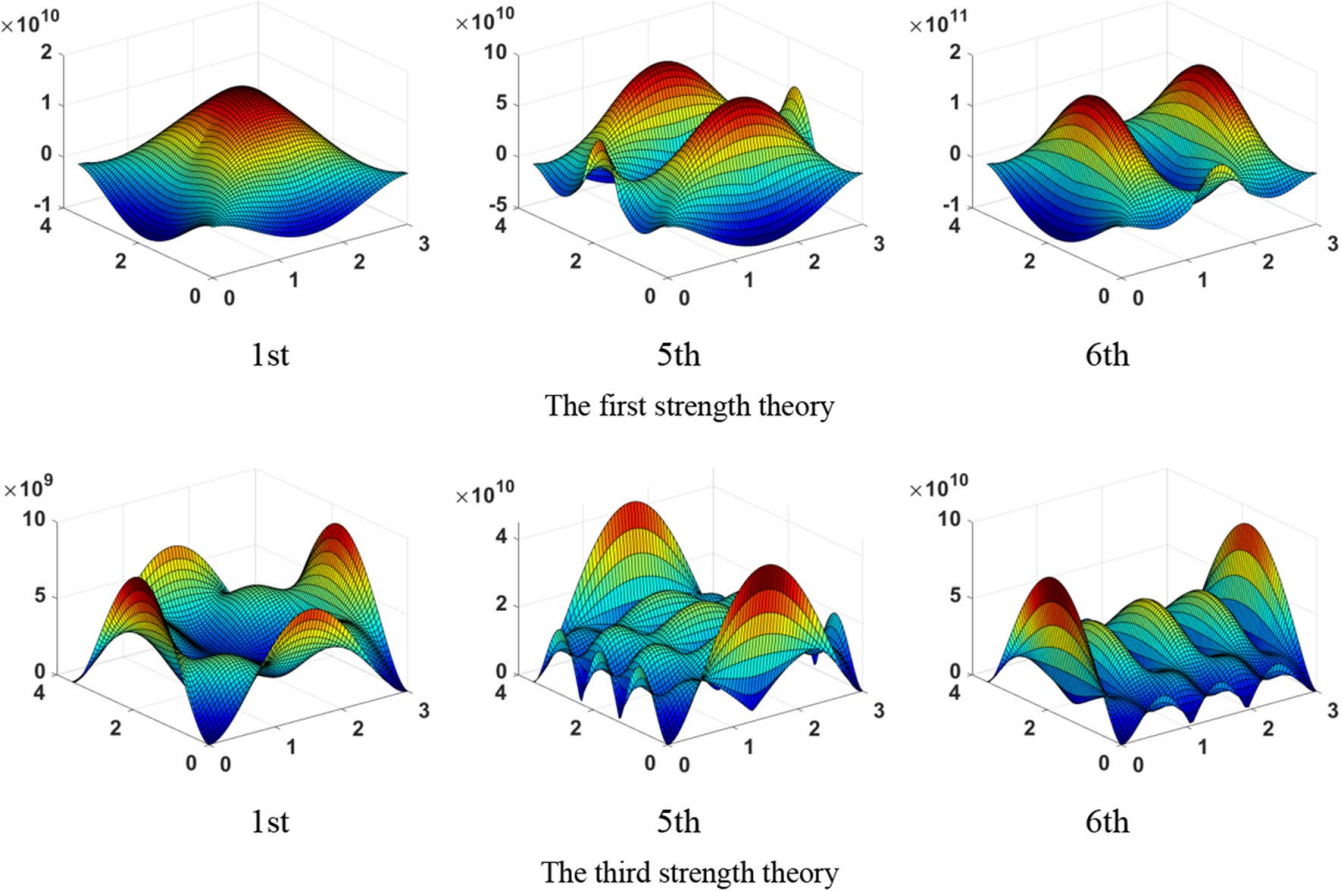
The maximum shear stress(τ_max_) distribution of the 1st, 5th and 6th mode.

Based on the first strength theory and third strength theory^66^, the maximum shear stress(τ_max_) distribution of the 1st, 5th and 6th mode as shown in Fig. 9. In 1st mode, the maximum shear stress concentrates at the middle of the four sides, which is symmetrical along the long and short central axis. The maximum shear stress of the 5th mode distributes in the long central axis and the middle of the two short sides, which are symmetrical along the long central axis. The maximum shear stress of the 6th mode distributes in the short central axis and the middle of the two long sides, which are symmetrical along the short central axis. It’s found that the stress *σ*_x_ of the 1st and 6th effective mode are greater than the stress *σ*_y_, the stress *σ*_y_ of the 5th effective mode is greater than the stress *σ*_x_, the shear stress is relatively small. So that the tensile failure is the main failure pattern in the center of thin plate rock mass. At the middle of the four sides on the thin plate rock mass, the stress *σ*_x_, *σ*_y_ and *τ*_max_ concentrate together. The shear stress *τ*_xy_ of the 1st, 5th and 6th modes and stress *σ*_y_ of the 6th mode are concentrated at four corners. Therefore, four sides of the thin plate rock mass are the tensile-shear failure caused by the combined action of tensile stress and shear stress, four corners are the shear failure caused by the shear stress. On the area outside the four sides and four corners, τ_max_ of the 1st mode is relatively small, τ_max_ of the 5th and 6th modes concentrate along the long central axis and short central axis of the thin plate respectively. When thin plate rock mass subjected to dynamic load, many main cracks are formed along the long central axis, a small number of secondary cracks are formed along the short central axis of thin plate.

#### Numerical simulation of the dynamic damage and failure

**Figure 10:**
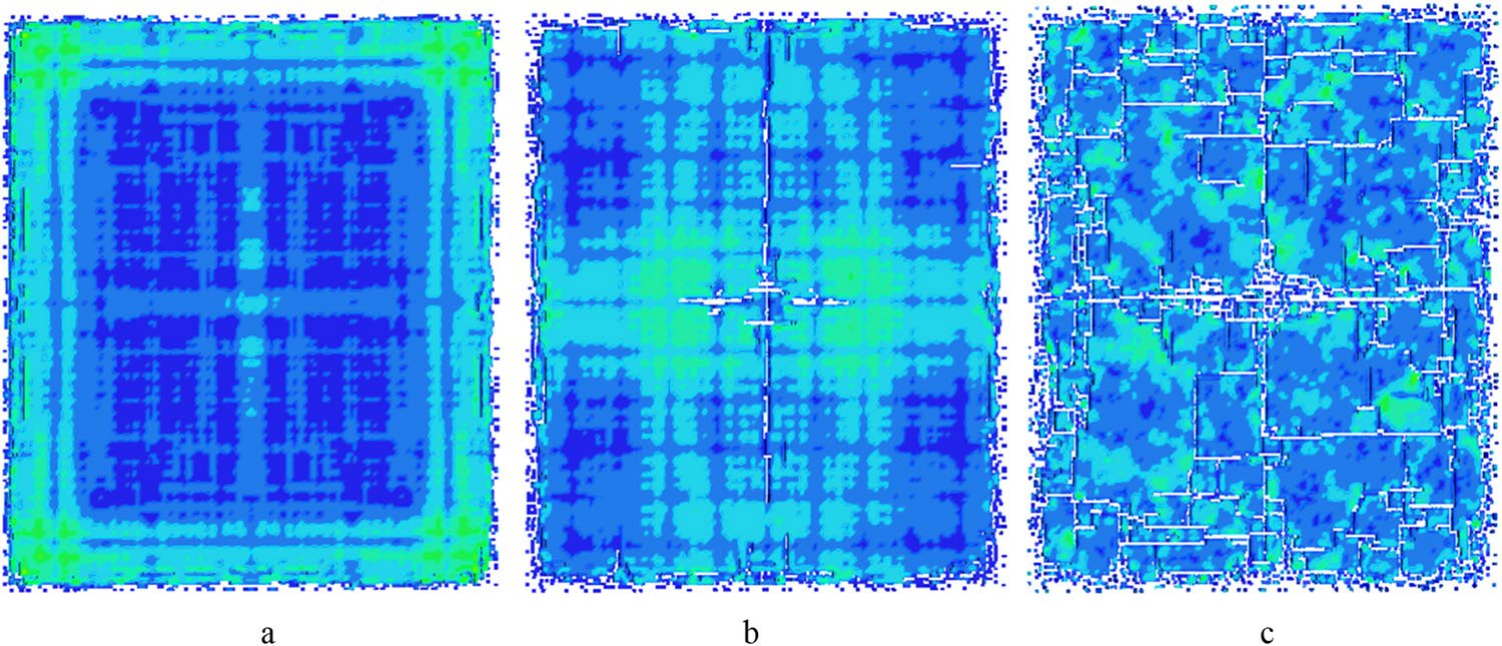
The dynamic damage and failure process of single-layered thin plate rock mass.

In LS-DYNA, the PLASTIC_KINEMATIC material model and Cowper Symonds model are used to simulate the failure of the thin plate rock mass under the dynamic load^67^. The dynamic damage and failure process of thin plate rock mass as shown in Fig. 10. From Fig. 10a, it’s found that cracks first appear in the middle and corners of the four sides on the thin plate, these cracks develop further along the boundary. Subsequently, cracks are generated in the center of the thin plate, the cracks extend outward along the long and short central axes of the thin plate in Fig. 10b. And the number of cracks along the long central axis is much larger than the cracks along the short central axis in Fig. 10c. The numerical simulation results are consistent with theoretical derivation in Fig. 9. So that, the failure position can be determined by 1st effective mode which in the middle of the four sides, four corners and central area of the thin plate. The trend of the cracks can be determined by the 5th and 6th effective modes, which along the long central axis and short central axis.

## Discussions

**Figure 11:**
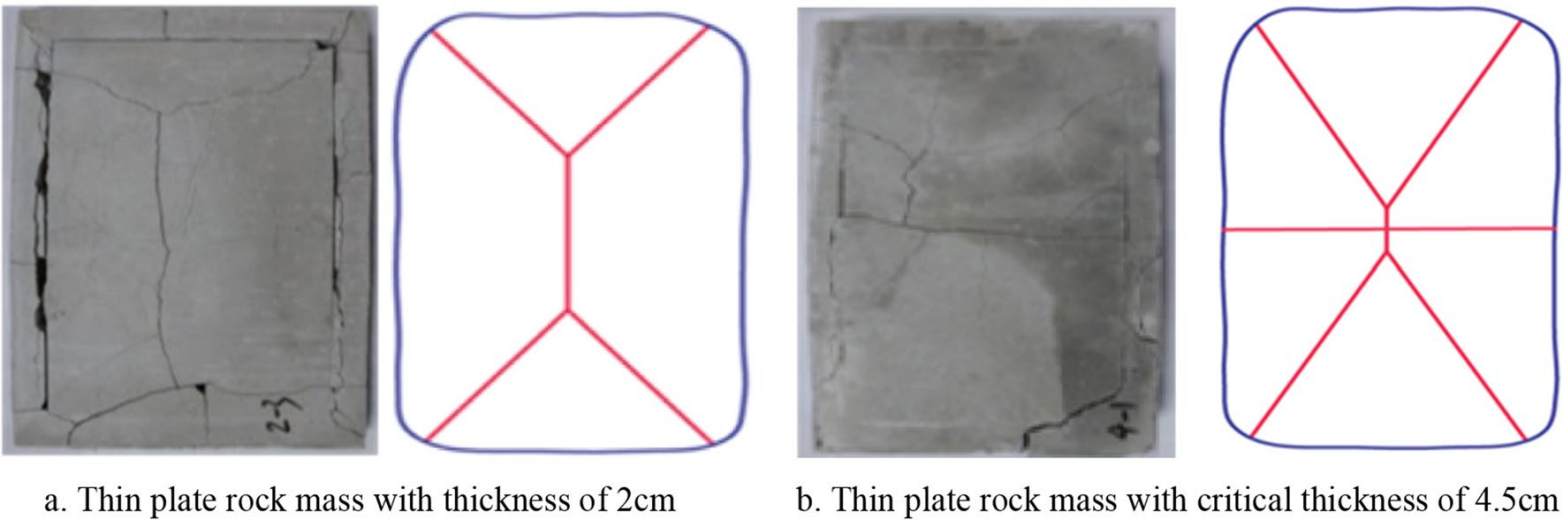
The failure pattern of single-layered thin plate rock mass under static load.

The failure patterns of single-layered thin plate rock mass with thickness of 2 cm and critical thickness of 4.5 cm were studied under static load by the simulation experiment device for roof breakage in goaf as shown in Fig. 11^44^. The results showed that: (1) With static load in Fig. 11a, the four sides of single-layered thin plate rock mass were subjected to the combined action of shear stress and tensile stress. The shear stress caused tensile shear failure on the four sides and formed an “O” ring. (2) The tensile failure not only occurred in the central area of single-layered thin plate rock mass, but also formed main vertical crack which propagated along the long axis. The main vertical crack splitted into “X” type crack, when it extended to the four corners. Finally, the “O-X” failure pattern was formed. (3) With the thickness of rock mass increases(critical thickness is 4.5 cm), it’s quite clear that there has a horizontal crack along the short central axis, which made the thin plate rock mass formed “O-❋” failure pattern in Fig. 11b.

According to the Figs. 9 and 10, four sides and four corners of single-layered thin plate rock mass occur tensile shear failure and shear failure under dynamic load. The main crack along the long central axis and the secondary crack along the short central axis are formed in the center of thin plate due to tensile-shear failure and shear failure. So the “O-**十**” failure pattern of single-layered thin plate rock mass is formed. Therefore, it’s obvious that the fracture mechanism of thin plate rock mass under dynamic load and static load is different, and the fracture characteristics are also different.The reason for the difference of fracture characteristics in thin plate rock mass is the stress loading condition in different ways. In Zuo’s study, the stress is mainly loaded to the rock mass under static loading conditions with low strain rate^12–14,44^, the failure mechanism is like the rock failure criterion which established by other scholars. However, the main content of this paper is the failure characteristics of layered rock under dynamic impact load with high strain rate. Based on the vibration characteristics, the deflection equation and effective vibration mode are deduced by Hamilton mechanical system. Combined with experiment and numerical simulation, the failure of single-layer thin rock mass is caused by the resonance which is induced by the effective vibration mode under dynamic impact load. On the one hand, the research results can use the resonance effect to accelerate the rock breaking process, reduce the energy consumption of rock breaking and improve the rock breaking efficiency; on the other hand, it can provide new ideas for the use and maintenance of built roadways and tunnels.

## Conclusions

In this paper Hamiltonian mechanics system is used to solve the deflection equations of single-layered thin plate rock mass, the main vibration modes and resonance characteristics under different dynamic loads can be obtained. Through theoretical analysis and numerical simulation, the failure mode of single-layered thin plate rock mass is obtained. The specific conclusions are as follows:

1. Based on the dual equation and Duhamel's integral, the mechanical model of single-layered thin plate rock mass is established. The main vibration modes (1st, 5th, and 6th modes) and the frequencies of thin plate rock mass are verified.
2. The resonance frequency interval and resonant amplitude φ_m_(t) caused by rectangular wave, triangular wave and impact wave can be obtained. With resonance frequency increase, the amplitude of resonance initiated by rectangular wave, triangular wave decreases exponentially, the amplitude of resonance initiated by impact wave keeps constant.
3. The thin plate is most likely to resonate and damage due to the smallest resonance frequency interval and the largest vibration amplitude by impact wave and rectangular wave respectively.
4. According to the MRIT, the vibration waveform can be decomposed into five vibration modes, the main vibration mode and the effective vibration modes are determined by energy analysis. Comparing main vibration modes parameters, it’s concluded the rock mass failure is caused by effective vibration modes of dynamic load.
5. Combining the first and third strength theory, it’s found that the failure of thin plate at the central area, four corners and the middle of the four sides, which determined by tensile stress and shear stress of 1st mode. Main cracks and secondary cracks formed along the long axis and short axis, which determined by τ_max_ of 5th and 6th modes.
6. Through numerical simulation, when tensile failure occurs in the center of thin plate rock mass, the cracks expand along the long axis and the short axis respectively to become the main crack and the secondary crack. finally the "O-**十**" failure pattern is formed which is different from the failure pattern formed by static load.

## Data Availability

ThE data used to support the findings of this study are available from the corresponding authors upon request.

## References

[1] Drzewiecki J, Myszkowski J (2018). Mining-induced seismicity of a seam located in rock mass made of thick sandstone layers with very low strength and deformation parameters. J. Min. Sci..

[2] Zhang T, Xu WY, Huang W, Wu GY (2020). Experimental study on mechanical properties of multi-layered rock mass and statistical damage constitutive model under hydraulic-mechanical coupling. Eur. J. Environ. Civ. Eng..

[3] Li A, Shao G, Su J, Liu J, Sun Y (2018). Uniaxial compression test and numerical simulation for alternatively soft and hard interbedded rock mass. Hehai Daxue Xuebao..

[4] Deng DP, Li L (2019). Failure modes and a calculation method for a stability analysis on a layered slope with a focus on interlayer sliding. Arabian J. Geosci..

[5] Zhou YY, Feng XT, Xu DP, Fan QX (2017). An enhanced equivalent continuum model for layered rock mass incorporating bedding structure and stress dependence. Int. J. Rock Mech. Min. Sci..

[6] Singh JG, Upadhyayxx PC (1987). Large elastic deflection of a rock plate. Min. Sci. Technol..

[7] Wang SY, Sloan SW, Tang CA, Zhu WC (2012). Numerical simulation of the failure mechanism of circular tunnels in transversely isotropic rock masses. Tunn. Undergr. Space Technol..

[8] Xu DP, Feng XT, Chen DF, Zhang CQ, Fan QX (2017). Constitutive representation and damage degree index for the layered rock mass excavation response in underground openings. Tunn. Undergr. Space Technol..

[9] Li A, Dai F, Liu Y, Du H, Jiang R (2021). Dynamic stability evaluation of underground cavern sidewalls against flexural toppling considering excavation-induced damage. Tunn. Undergr. Space Technol..

[10] Li JC, Wu W, Li HB, Zhu JB, Zhao J (2013). A thin-layer interface model for wave propagation through filled rock joints. J Appl Geophy..

[11] Latha GM, Garaga A (2012). Elasto-plastic analysis of jointed rocks using discrete continuum and equivalent continuum approaches. Int. J. Rock Mech. Min. Sci..

[12] Zuo J, Chen Y, Cui F (2018). Investigation on mechanical properties and rock burst tendency of different coal-rock combined bodies. Zhongguo Kuangye Daxue Xuebao.

[13] Chen Y, Zuo J, Song H, Feng L, Shao G, Beijing T (2018). Deformation and crack evolution of coal-rock combined body under cyclic loading-unloading effects. Kuangshan Yali..

[14] Yan C, Zuo J, Xu W, Song H, Sun Y (2017). Energy nonlinear evolution characteristics of the failure behavior of coal-rock combined body. Dixia Kongjian.

[15] Zuo JP, Chen Y, Song HQ, Xu W (2017). Evolution of pre-peak axial crack strain and nonlinear model for coal-rock combined body. Yantu Gongcheng Xuebao..

[16] Zuo JP, Chen Y, Zhang JW, Wang JT, Jiang GH (2016). Failure behavior and strength characteristics of coal-rock combined body under different confining pressures. Meitan Xuebao..

[17] Wang GZ, Wang Y, Song L, Shi H, Zhang MW, Yuan GT (2021). Particle flow simulation of the strength and failure characteristics of a layered composite rock-like sample with a single hole. Symmetry-Basel..

[18] Liu XF, Feng XT, Zhou YY, Sharifzadeh M (2022). Influences of foliation orientation and lateral stress difference on the deformation and fracturing of a thin-layered rock around underground excavations: insight from multi-axial loading tests. Bull. Eng. Geol. Environ..

[19] Cai M (2008). Influence of intermediate principal stress on rock fracturing and strength near excavation boundaries - Insight from numerical modeling. Int. J. Rock Mech. Min. Sci..

[20] Zienkiewicz OC, Pande GN (2010). Time-dependent multilaminate model of rocks—a numerical study of deformation and failure of rock masses. Int. J. Numer. Anal. Methods Geomech..

[21] Wu B, Jia SP, Luo JZ (2015). Anisotropic composite model for layered rock mass based on characteristics of soft interfaces. Mater. Res. Innov..

[22] Zhao N, Zhang YB, Miao HB, Meng LX (2022). Study on the creep and fracture evolution mechanism of rock mass with weak interlayers. Adv. Mater. Sci. Eng..

[23] Wang HT, Liu P, Li SC, Li XJ, Zhang X (2022). Limit analysis of uplift failure mechanisms for a high-pressure gas storage tunnel in layered Hoek-Brown rock masses. Eng. Fail Anal..

[24] Mohammadi M, Hossaini MF (2017). Modification of rock mass rating system: interbedding of strong and weak rock layers. J. Rock Mech. Geotech. Eng..

[25] Shi LK, Zhou H, Song M, Lu JJ, Liu ZJ (2021). Geomechanical model test for analysis of surrounding rock behaviours in composite strata. J. Rock Mech. Geotech. Eng..

[26] He JZ, Li YA, Jin YL, Wang AM, Zhang YM, Jia JC (2022). Study on mechanical problems of complex rock mass by composite material micromechanics methods: a literature review. Front. Earth Sci..

[27] Li SH, Zhu WC, Niu LL, Yu M, Chen CF (2018). Dynamic characteristics of green sandstone subjected to repetitive impact loading: phenomena and mechanisms. Rock Mech. Rock Eng..

[28] Chai SB, Li JC, Rong LF, Li NN (2017). Theoretical study for induced seismic wave propagation across rock masses during underground exploitation. Geomech. Geophys. Geo..

[29] Feng F, Li XB, Li DY, Wang SF (2017). Mechanism and control strategy of buckling rockbursts of orthotropic slab. Yantu Gongcheng Xuebao..

[30] Liu DQ, Ling K, Li D, He MC, Li JY, Han ZJ (2021). Evolution of anisotropy during sandstone rockburst process under double-faces unloading. J. Cent. South Univ. Technol. (Engl. Ed.).

[31] Tian Y, Chen WZ, Tian HM, Yang JP, Zhang ZY, Shu XY (2021). Analytical model of layered rock considering its time-dependent behaviour. Rock Mech Rock Eng..

[32] Zhou T, Zhu JB, Xie HP (2020). Mechanical and volumetric fracturing behaviour of three-dimensional printing rock-like samples under dynamic loading. Rock Mech. Rock Eng..

[33] Wang J, Chen G, Xiao Y, Li S, Qiao Z (2021). Effect of structural planes on rockburst distribution: case study of a deep tunnel in Southwest China. Eng Geol..

[34] Braunagel MJ, Griffith WA (2019). The effect of dynamic stress cycling on the compressive strength of rocks. Res. Lett..

[35] Xie B, Yan Z (2019). Dynamic mechanical constitutive model of combined coal-rock mass based on overlay model. Meitan Xuebao..

[36] Wen S, Zhang CS, Chang YL, Hu P (2020). Dynamic compression characteristics of layered rock mass of significant strength changes in adjacent layers. J. Rock Mech. Geotech. Eng..

[37] Han ZY, Li DY, Zhou T, Zhu QQ, Ranjith PG (2020). Experimental study of stress wave propagation and energy characteristics across rock specimens containing cemented mortar joint with various thicknesses. Int. J. Rock Mech. Min. Sci..

[38] Han ZY, Li DY, Li XB (2022). Dynamic mechanical properties and wave propagation of composite rock-mortar specimens based on SHPB tests. Int. J. Min. Sci. Technol..

[39] Qiu H, Wang F, Zhu ZM, Wang M, Yu DM, Luo CS (2021). Study on dynamic fracture behaviour and fracture toughness in rock-mortar interface under impact load. Compos. Struct..

[40] Qiu H, Zhu ZM, Wang F, Wang M, Zhou CL, Luo CS (2020). Dynamic behavior of a running crack crossing mortar-rock interface under impacting load. Eng. Fract. Mech..

[41] Zheng KH, Qiu BJ, Wang ZY, Li JP, Gao KD (2020). Modelling heterogeneous coal-rock (HCR) failure patterns under dynamic impact loads using image-based finite element (FE) and discrete element (DE) model. Powder Technol..

[42] Zheng KH, Qiu BJ, Wang ZY, Li XF, Li JP, Gao KD (2020). Image-based numerical study of three-dimensional meso-structure effects on damage and failure of heterogeneous coal-rock under dynamic impact loads. Particuology..

[43] Duan JZ, Zhou SW, Xia CC, Xu YJ (2023). A dynamic phase field model for predicting rock fracture diversity under impact loading. Int. J. Impact Eng..

[44] Zuo J, Yu M, Hu S, Song H, Wei X, Shi Y (2019). Experimental investigation on fracture mode of different thick rock strata. J. Min. Sci..

[45] Yao W, Yang H (2001). Hamiltonian system based Saint Venant Solutions for multi-layered compostie plane anisotropic plates. Int. J. Solids Struct..

[46] Zhong W, Yao W (1997). The Saint Venant solutions of multi-layered composite plates. Adv. Struct. Eng..

[47] Xu XS, Zhong WX, Zhang HW (1997). The Saint-Venant problem and principle in elasticity. Int. J. Solids Struct..

[48] Zhao L, Chen WQ, Lu CF (2012). New assessment on the Saint-Venant solutions for functionally graded beams. Mech Res Commun..

[49] Zhong W, Xu X, Zhang H (1996). Hamiltonian system and the saint venant problem in elasticity. Appl. Math. Mech-Engl..

[50] Zhong Y, Li R, Tian B (2011). Hamiltonian analytical solution offree vibration of rectangular thin plate with four sides fixed. Yingyong Lixue Xuebao..

[51] Zhong W (1994). Plane elasticity in sectorial domain and the Hamiltonian system. Appl. Math. Mech-Engl..

[52] Wanxie Z, Jiahao L, Chunhang Q (1992). Computational structural mechanics and optimal control—the simulation of substructural chain theory to linear quadratic optimal control problems. Int. J. Numer. Methods in Eng..

[53] Leung AYT, Zheng JJ, Lim CW, Zhang XC, Xu XS, Gu Q (2008). A new symplectic approach for piezoelectric cantilever composite plates. Comput. Struct..

[54] Su X, Bai E (2022). Analytical free vibration solutions of fully free orthotropic rectangular thin plates on two-parameter elastic foundations by the symplectic superposition method. J. Vib. Control..

[55] Zhang, W X. IEEE: Dual method for problems of transversely isotropic elastic solids, In *2nd World Conference on Mechanical Engineering and Intelligent Manufacturing (WCMEIM)*, Shanghai Univ Engn Sci, Shanghai, Peoples R China, Nov 22–24, **2019**; pp 151–154 (2019).

[56] Li F, Zhang Y, Fang S, Zhang L, Liu J (2016). Dynamic damage characteristics of elastic and plastic combination coal under impact loading. Meitan Xuebao..

[57] Xing Y, Bo L (2009). New exact solutions for free vibrations of rectangular thin plates by symplectic dual method. Acta Mech Sin..

[58] Bao S (2005). A general solution of free vibration for rectangular thin plates in Hamilton systems. J. Dyn. Control..

[59] Bao S, Deng Z (2016). High-precision approximate analyticalsolutions for free bending vibrations of thin plates and an improve-ment. Appl. Math. Mech..

[60] Li R, Wang B, Li P (2014). Hamiltonian system-based benchmark bending solutions of rectangular thin plates with a corner point-supported. Int. J. Mech. Sci..

[61] Yang G, Lin C (2005). Forced vibration analysis of rectan-gular thin plates with mixed boundary. J. Chi. Feng Univ..

[62] Zhang J, Guo SL, Zhang QQ (2015). Mobile impact testing for structural flexibility identification with only a single reference. Comput-Aided Civ. Inf..

[63] Liu S, Rawat P, Zhu DJ (2022). Understanding the effects of altering impact velocities and temperatures on basalt textile: an experiment approach. Int. J. Impact Eng..

[64] Brown DL, Witter MC (2011). Review of recent developments in multiple-reference impact testing. Sound Vib..

[65] Khader, N.;Ramadan, M: Modal parameters of multiple-disk shaft system from multiple reference impact test. In *34th IMAC Conference and Exposition on Structural Dynamics*, Orlando, FL, Jan 25–28, **2016**; pp 67–77 (2016).

[66] Li F, Hu Z, Chen N, Zhang Y, An X (2022). A study of fracture range of tunnel surrounding rock under blasting. Shock. Vib..

[67] Hu R, Zhu Z, Hu Z (2013). Experimentalstudy of regularity of crack prppagation under blastingdynamic loads. Yanshi Yanshi Lixue Yu Gongcheng Xuebao..

